# Diagnostic Efficiency in Urinary Tract Infection: Evaluation of a Comprehensive Intervention Based on Clinical Conditions, Urinalysis, and Gram Staining

**DOI:** 10.7759/cureus.100750

**Published:** 2026-01-04

**Authors:** Carlos Solórzano, Maria Camila Rubio, Jeimy Lorena León, Maria Alejandra Caro, Luis Alejandro Olarte Licht, Juan Manuel Luna Gualdron, Edgar F Manrique-Hernandez, Maricel Licht-Ardila, Alexandra Hurtado Ortiz

**Affiliations:** 1 Department of Infectious Diseases, Hospital Internacional de Colombia, Fundación Cardiovascular de Colombia, Piedecuesta, COL; 2 Department of Infectious Diseases, Facultad de Medicina, Universidad El Bosque, Bogotá, COL; 3 Department of General Medicine, Facultad de Medicina, Universidad Autónoma de Bucaramanga, Bucaramanga, COL; 4 Department of Epidemiology, Fundación Cardiovascular de Colombia, Floridablanca, COL; 5 Department of Epidemiology and Public Health, Fundación Cardiovascular de Colombia, Piedecuesta, COL; 6 Department of Epidemiology and Public Health, Hospital Internacional de Colombia, Fundación Cardiovascular de Colombia, Piedecuesta, COL; 7 Postgraduate Department in Infectious Disease, Universidad de Santander, Santander, COL

**Keywords:** antimicrobial stewardship, bacteriuria, diagnostic tests, leukocyte esterase, routine, urinary tract infections

## Abstract

Introduction: Urinary tract infection (UTI) is the second most common cause of infection globally, following respiratory system infections. The aim is to evaluate the effectiveness of an optimization strategy in the diagnosis of UTIs through the patient’s clinical condition and laboratory tests, with the purpose of reducing unnecessary urine culture processing, reducing antibiotic treatment of asymptomatic bacteriuria, and improving the diagnostic accuracy of UTIs.

Methods: A quasi-experimental study was conducted in a high-complexity hospital’s emergency department from January to September 2024. Adult patients with suspected UTIs were included. The intervention, implemented in April, consisted of a protocol created by the Antimicrobial Stewardship Program for diagnostic accuracy of UTIs. Data on sociodemographic, clinical, laboratory, and cost-related variables were analyzed.

Results: A total of 1,376 urine cultures were included. The intervention showed a statistically significant (p < 0.001) decrease in the processing of urine culture in the intervention and postintervention group, with 71% and 84% less urine cultures processed than the preintervention group, respectively. No differences were found (p = 0.384) in readmission due to UTI within the next 30 days or in mortality (p = 0.488). The intervention reduced the number of urine cultures collected, leading to an 84.05% decrease in costs compared to the preintervention period.

Conclusion: The implementation of an optimized urine culture protocol that incorporates clinical conditions and laboratory tests can lead to a significant reduction in costs and waste, while also promoting better optimization of available resources.

## Introduction

Urinary tract infection (UTI) is the second most common cause of infection globally, following respiratory system infections [1]. The assessment and treatment of UTIs involve significant costs worldwide. One study showed that in the United States, the average cost of hospital visits for UTI over seven consecutive years was $7,766, increasing to $9,902 if the infection was caused by a resistant pathogen [2]. In Colombia, the prevalence of UTIs varies across different population groups. In Medellín, a study conducted in adult patients revealed a prevalence of 31% at a healthcare institution in the general population [3]. In American hospitals within the Society for Healthcare Epidemiology of America network, a diagnostic evaluation based on urine cultures was conducted, revealing that urine cultures are commonly performed in asymptomatic patients. Only 44% of institutions have clear guidelines for urine culture collection, and just 17% assess symptoms before proceeding with urine testing [4].

The accurate diagnosis of UTIs has been a challenge over time, as it requires the correlation between generally nonspecific symptoms and various laboratory results. Urinalysis has demonstrated good performance, as both leukocyte esterase and nitrite detection have diagnostic accuracy for predicting the absence of bacteriuria compared with urine culture, together constituting a likelihood ratio greater than 9. Similarly, uncentrifuged urine Gram staining has high sensitivity for identifying urine culture positivity, showing a negative predictive value of 97% for significant bacteriuria [5]. It is estimated that the combination of these parameters allows for the accuracy of urine culture processing, which not only represents a significant increase in healthcare costs but may also lead to unnecessary treatments due to asymptomatic bacteriuria that ultimately contribute to the promotion of antimicrobial resistance [6].

The identification of significant bacterial growth in a urine sample in the absence of symptoms is referred to as asymptomatic bacteriuria [7]. While antibiotic treatment is beneficial for patients with urinary symptoms, asymptomatic bacteriuria should not be treated except in specific scenarios, such as in pregnant women and patients undergoing urological procedures [8]. Therefore, optimizing the diagnosis and management of UTI is crucial to reduce both the unnecessary use of resources and the risk of developing antimicrobial resistance [9].

The Antimicrobial Stewardship Program is an initiative created in response to the increasing antimicrobial resistance, with the goal of optimizing clinical outcomes, reducing adverse events, and decreasing the costs associated with the use of antimicrobials [10]. Additionally, this program may include the implementation of standardized microbiology diagnostic stewardship strategies, including the development of structured diagnostic algorithms for infectious diseases [11]. Within this framework, a standardized rational urine culture protocol was developed and implemented, integrating predefined clinical criteria and laboratory parameters to guide urine culture processing and avoid unnecessary testing.

Given this, the aim is to evaluate the effectiveness of a rational urine culture protocol, created by the Antimicrobial Stewardship Program of a high-complexity hospital in Colombia. Secondary outcomes included a reduction in unnecessary urine culture processing based on patients’ clinical conditions and laboratory tests, improvements in the diagnostic accuracy of UTIs, and associations with clinical outcomes such as hospital readmission and mortality, as well as economic outcomes related to diagnostic and antimicrobial-related costs.

## Materials and methods

A quasi-experimental study was conducted, including patients who attended the emergency department of a high-complexity hospital in Colombia from January 1 to September 30, 2024. The inclusion criteria comprised adult patients (≥18 years) with suspected UTI who underwent diagnostic tests, such as urine culture, Gram stain, and urinalysis, during their stay in the emergency department. Patients admitted after April 5 who did not undergo simultaneous urine Gram stain, urinalysis, and urine culture were excluded, except for pregnant patients and those requiring urological procedures, defined as any procedure that breaches the mucosa of the urinary tract. Additionally, cases with incomplete records were also excluded.

The intervention involved implementing a protocol developed by the Antimicrobial Stewardship Program to improve diagnostic accuracy for UTIs. In the beginning, a digital form was designed for use by emergency department physicians. This standardized digital form was integrated into the emergency department clinical workflow and guided physicians in real time during decision-making regarding requests for urine cultures. This form, structured around a series of specific questions, aimed to guide physicians in determining the need for a urine culture based on the responses provided. Additionally, a microbiology diagnostic stewardship was implemented by developing a diagnostic algorithm based on the results of urinary diagnostic tests, including urinalysis and Gram stain. The protocol was introduced on April 5, 2024. Patients seen during the first quarter of 2024, before the protocol implementation, comprised the preintervention group. Patients seen during the second quarter of 2024, when the protocol was actively used by physicians and the microbiology laboratory, formed the intervention group. Additionally, patients seen between July 1 and September 30, 2024, were included in the postintervention group. Patients with asymptomatic bacteriuria were defined as those who exhibited none of the assessed UTI symptoms during data collection.

This protocol, for the rational request of urine cultures, considers whether the patient is pregnant or a candidate for any urological procedure. Suppose the patient meets either one of these criteria, a urine culture should be requested. Otherwise, urinary irritative symptoms are assessed, including dysuria, frequency, urgency, suprapubic pain, hematuria, fever, and costovertebral pain. For processing the urinary diagnostic tests, the following criteria were required: in young women, the presence of at least three of the five assessed urinary symptoms; in postmenopausal women, the presence of any urinary symptom; in men, the presence of any urinary symptom; in both men and women, the presence of fever and/or costovertebral angle pain; and in patients with a urinary catheter, the presence of fever and costovertebral angle pain.

**Figure 1:**
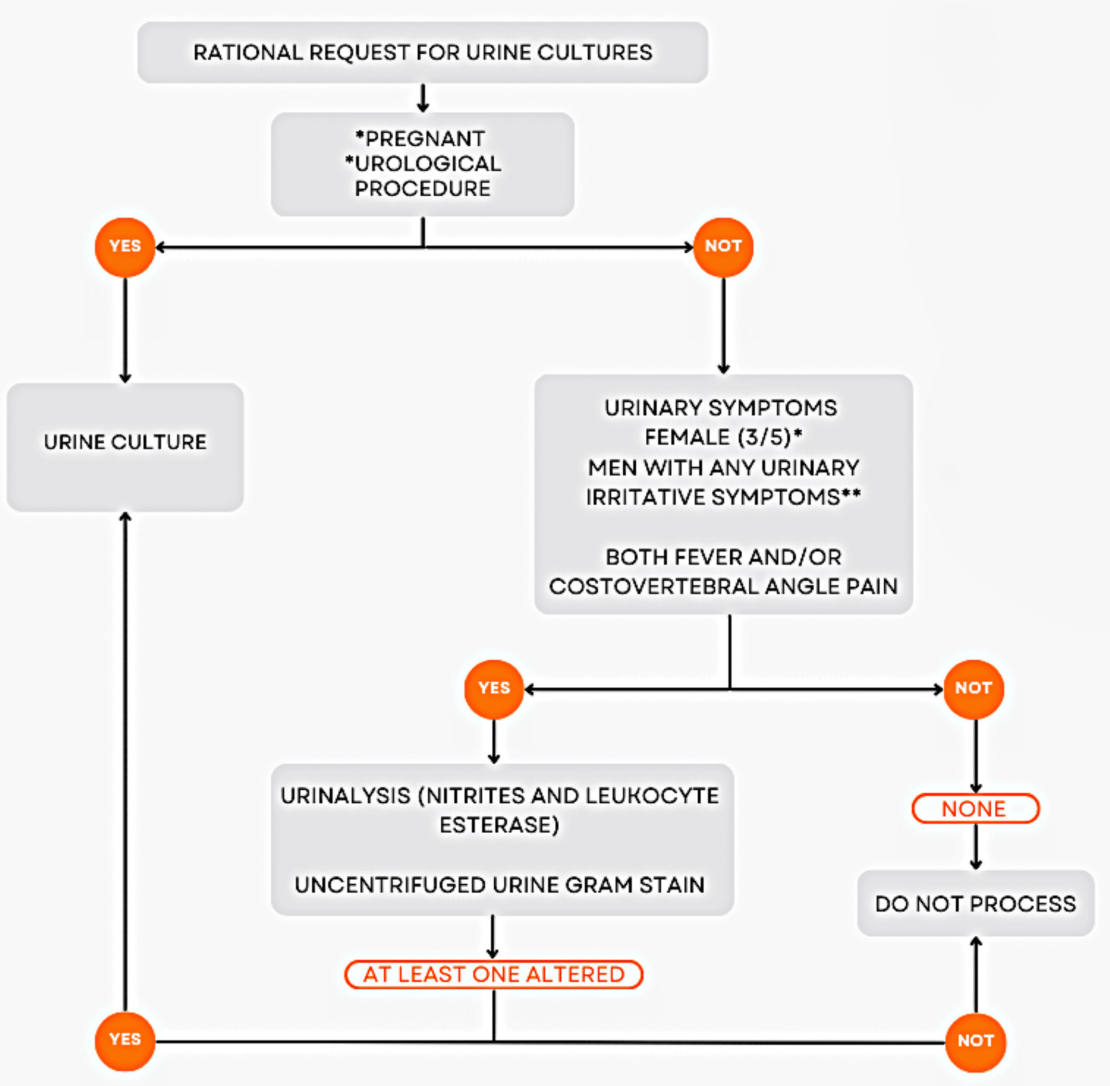
Flowchart for rational request and processing of urine cultures in the emergency department ^*^Symptom-based criteria applied to women (greater than or equal to three of five urinary symptoms in young women or any urinary symptom in postmenopausal women) ^**^Symptom-based criteria applied to men (any urinary irritative symptom)

If symptoms for each group are not present, the digital form suggests to the physician: do not test for UTI. If symptoms are present, the digital form lets you order the urinary diagnostic tests, including urinalysis, urine Gram stain, and urine culture. Then, microbiologists perform the diagnostic algorithm. They perform urinalysis and Gram staining on uncentrifuged urine specimens [12]. First, if any results are abnormal, a urine culture is processed (Figure 1). We defined a positive result of Gram stain as the presence of at least one bacterium per high-level field. For the urinalysis (nitrites and positive esterase): conducted using the COMBUR 10 Test M by Cobas® (Roche Diagnostics, Germany), this analysis focuses on detecting nitrites with a detection limit of 0.07 mg/dL and leukocyte esterase with a detection limit of 12-25 leukocytes per microliter. The urine cultures are performed using inoculation on MacConkey Agar and Blood Agar. A culture is considered positive when a single organism is isolated at concentrations ≥105 colony-forming units per milliliter.

Data collection

Patient data were extracted from the institutional electronic health record system and the Infectious Diseases Department database, both of which were anonymized prior to analysis. The variables collected included sociodemographic, clinical, laboratory, and cost-related data.

Sociodemographic variables encompassed age, health insurance provider, healthcare system affiliation (contributory, subsidized, or other), geographic area of residence, and sex. Clinical variables included a history of prior urological procedures, nonurological surgeries, diabetes mellitus, rheumatologic diseases, HIV, benign prostatic hyperplasia, urolithiasis, urinary tract neoplasms, kidney transplantation, hematopoietic stem cell transplantation, use of biological therapy, use of urinary devices, and use of antibiotics in the past 90 days. Additionally, data were collected on pregnancy status, symptoms such as dysuria, polyuria, urgency, suprapubic pain, hematuria, fever, and costovertebral pain, as well as clinical complications including diarrhea, chemical phlebitis, duration of antibiotic regimens, recent hospitalizations, readmissions due to UTIs within the last 30 days, and nonurological surgeries.

Laboratory variables included urine culture results, microorganism identification, leukocyte esterase test results, nitrite test results, urine Gram stain results, and creatinine levels. Cost-related variables encompassed cost data.

Statistical analysis

Descriptive statistics were used to summarize patient demographics, clinical characteristics, and laboratory findings. Continuous variables were expressed as mean ± standard deviation or median with quartiles (Q1-Q3), depending on their distribution, which was assessed using the Shapiro-Wilk test. Categorical variables were presented as frequencies and percentages. Comparisons between groups (preintervention vs. intervention vs. postintervention) were conducted using the Kruskal-Wallis test for continuous variables and chi-square or Fisher’s exact tests for categorical variables.

A statistical modeling analysis was conducted to study the relationship between the urine cultures performed and the number of urine cultures avoided. Additionally, the costs associated with each of these procedures were assessed, thus enabling a deeper understanding of the generated benefits and efficiencies. All statistical analyses were conducted using Stata version 17 (StataCorp LLC, College Station, TX) [13].

Ethical considerations

The study was approved by the institutional ethics committee with code CEI-2024-07-461-4. All patient data were anonymized to ensure confidentiality, and the study adhered to the principles outlined in the Declaration of Helsinki.

## Results

**Figure 2:**
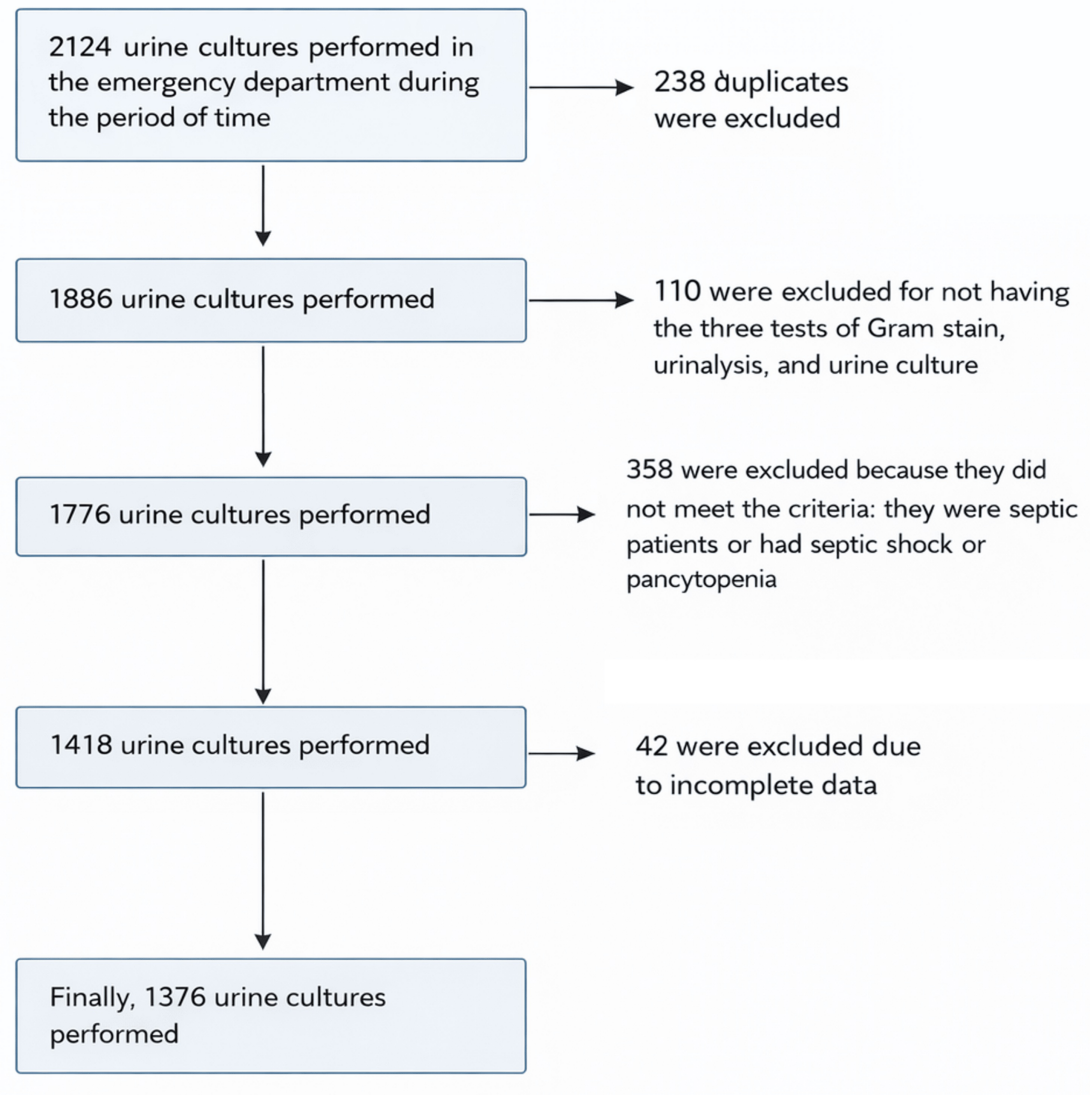
Flowchart of cohort selection

A total of 1,376 urine cultures (100%) were collected from the emergency department of a tertiary care hospital, which were included in the study (Figure 2). In the analysis of care provided, a 7.3 percentage-point increase in the total number of attentions was observed during the intervention compared with the no-intervention group. On the other hand, comparing the postintervention group with the intervention group revealed a 9.8 percentage-point decrease. The median age of patients was 58 years (Q1-Q3 = 40-76) overall, with no statistically significant differences between groups: preintervention 58 years (IQR = 40-45), intervention 59 years (Q1-Q3 = 39-73), and postintervention 62 years (Q1-Q3 = 44-76) (p = 0.597). The sex distribution showed no significant differences (p = 0.488), with 400 (42.24%) of male patients in the preintervention phase (Table 1).

**Table 1:** Baseline characteristics of patients across preintervention, intervention, and postintervention groups

Variable	Category	Preintervention, n = 947 (%)	Intervention, n = 278 (%)	Postintervention, n = 151 (%)	Total, n = 1,376 (%)	p value
Sex	Female	547 (57.76)	177 (63.67)	89 (58.94)	813 (59.08)	0.488
	Male	400 (42.24)	101 (36.33)	62 (41.06)	563 (40.92)	
Healthcare regimen	Subsidized	382 (40.34)	98 (35.25)	63 (41.72)	543 (39.46)	0.202
	Contributory	437 (46.15)	132 (47.48)	61 (40.4)	630 (45.78)	
	Other	128 (13.52)	48 (17.27)	27 (17.88)	203 (14.75)	
Area of residence	Rural	140 (14.78)	54 (19.42)	25 (16.56)	219 (15.92)	0.173
	Urban	807 (85.22)	224 (80.58)	126 (83.44)	1,157 (84.08)	

A history of hospitalization within the last 90 days was reported in 363 (38.91%) of patients in the preintervention phase, increasing to 126 (48.28%) during the intervention, and remaining at 74 (49.01%) postintervention (p = 0.004). The frequency of kidney transplantation was low in all groups but increased from three (0.32%) preintervention to five (1.92%) during the intervention, before decreasing to one (0.66%) postintervention (p = 0.020). Conversely, hematopoietic stem cell transplantation was rare, with only one (0.11%) of cases preintervention, 0% during the intervention, and an increase to three (1.99%) postintervention (p < 0.001).

The use of biological therapy was significantly different between groups: 25 (2.68%) preintervention, eight (3.07%) during the intervention, and 19 (12.58%) postintervention (p < 0.001). Urinary tract neoplasms increased from 27 (2.9%) preintervention to 19 (7.28%) and then to 21 (14%) postintervention (p < 0.001). Additionally, the use of antibiotics within the last 90 days was reported in 261 (28%) of patients preintervention, increasing to 102 (39.08%) during the intervention and then decreasing to 51 (33.77%) postintervention (p = 0.002) (Table 2). Overall, these findings indicate a progressive change in the clinical profile of the study population across the three periods, with an increasing proportion of patients receiving biological therapies and diagnosed with urinary tract neoplasms. In contrast, recent antibiotic exposure peaked during the intervention phase and subsequently declined, although remaining higher than preintervention levels.

**Table 2:** Medical history of patients across preintervention, intervention, and postintervention groups BPH: benign prostatic hyperplasia

Variable	Category	Preintervention, n = 947 (%)	Intervention, n = 278 (%)	Postintervention, n = 151 (%)	Total, n = 1,376 (%)	p value
Medical history						
Hospitalization within the last 90 days		363 (38.91)	126 (48.28)	74 (49.01)	563 (41.86)	0.004
Diabetes		158 (16.93)	51 (19.54)	21 (13.91)	230 (17.1)	0.333
Kidney transplantation		3 (0.32)	5 (1.92)	1 (0.66)	9 (0.67)	0.020
Hematopoietic stem cell transplantation		1 (0.11)	0 (0)	3 (1.99)	4 (0.3)	<0.001
Rheumatologic diseases		17 (1.82)	8 (3.08)	2 (1.32)	27 (2.01)	0.362
Use of biological therapy		25 (2.68)	8 (3.07)	19 (12.58)	52 (3.87)	<0.001
HIV		9 (0.97)	3 (1.15)	0	12 (0.89)	0.447
BPH		79 (8.47)	21 (8.05)	19 (12.58)	119 (8.85)	0.225
Urolithiasis		105 (11.25)	40 (15.33)	14 (9.27)	159 (11.82)	0.116
Prior urological procedures		111 (11.9)	60 (22.99)	53 (35.1)	224 (16.65)	<0.001
Urinary tract neoplasm		27 (2.9)	19 (7.28)	21 (14)	67 (4.99)	<0.001
Use of antibiotics within the last 90 days		261 (28)	102 (39.08)	51 (33.77)	414 (30.8)	0.002

Regarding leukocyte esterase, nitrites, and Gram stain analyses, significant differences were observed between groups. Leukocyte esterase and Gram stain were consistently positive across all groups, while nitrite was consistently negative in all phases. In the preintervention group, patients with asymptomatic bacteriuria represented 250 (26.8%) of the urine cultured processed, with a statistically significant decrease in the intervention and postintervention group (p = 0.001). Hospitalization percentage was also higher in the intervention 182 (65.47%) and postintervention 107 (70.86%) groups than in the preintervention phase, 514 (54.28%) (p < 0.001) (Table 3).

**Table 3:** Clinical and microbiological characteristics across the preintervention, intervention, and postintervention groups UTI: urinary tract infection

Variable	Preintervention, n = 947 (%)	Intervention, n = 278 (%)	Postintervention, n = 151 (%)	Total, n = 1,376 (%)	p value
Elective nonurological surgery	31 (3.32)	4 (1.53)	3 (1.99)	38 (2.83)	0.245
Emergency urological surgery	19 (2.04)	19 (7.28)	16 (10.6)	54 (4.01)	<0.001
Urological surgical procedure	39 (4.12)	21 (7.55)	10 (6.62)	70 (5.09)	0.048
Nonurological surgical procedure	76 (8.03)	23 (8.27)	10 (6.62)	109 (7.92)	0.814
Pregnant women	4 (0.43)	5 (1.92)	1 (0.66)	10 (0.74)	0.046
Hospitalization	514 (54.28)	182 (65.47)	107 (70.86)	803 (58.36)	<0.001
Asymptomatic bacteriuria	250 (26.80)	44 (16.86)	29 (19.21)	323 (24.01	0.001
Urine culture					
Negative	484 (51.11)	83 (29.86)	40 (26.49)	607 (44.11)	<0.001
Positive	463 (48.89)	195 (70.14)	111 (73.51)	769 (55.89)	
Leukocyte esterase					
Negative	298 (32.29)	39 (14.18)	23 (15.33)	360 (26.71)	<0.001
Positive	625 (67.71)	236 (85.82)	127 (84.67)	988 (73.29)	
Nitrites					
Negative	681 (73.78)	157 (57.09)	100 (66.67)	938 (69.58)	0.002
Positive	242 (26.22)	118 (42.91)	50 (33.33)	410 (30.42)	
Gram stain					
Negative	158 (17.67)	83 (32.81)	38 (26.95)	279 (21.66)	<0.001
Positive	736 (82.33)	170 (67.19)	103 (73.05)	1,009 (78.34)	
Readmission due to UTI within 30 days					
Yes	94 (10.09)	30 (11.54)	11 (7.28)	135 (10.05)	0.384
No	838 (89.91)	230 (88.46)	140 (92.72)	1,208 (89.95)	
Length of stay, median (Q1-Q3) (days)	3 (1-7)	4 (1-10)	5 (2-9)	3 (1-8)	0.002
In-hospital mortality	51 (5.39)	20 (7.19)	10 (6.62)	81 (5.89)	0.488

Concerning the isolated microorganisms, *Escherichia coli* was the most common pathogen, accounting for 387 (56.41%) of total cases. A decrease was observed in the intervention 55.17% and postintervention 49.02% groups (p = 0.091). Other relevant microorganisms included *Klebsiella pneumoniae*, 127 (18.51%), *Proteus mirabilis*, 35 (5.1%), and *Pseudomonas aeruginosa*, 34 (4.96%), with a similar distribution across groups (Table 4). In the analysis of antibiotics used to treat UTIs, ceftriaxone was the most frequently prescribed antibiotic; however, the distribution across the three groups did not show statistically significant differences (p = 0.086) (Table 5).

**Table 4:** Microorganisms isolated from urine cultures across the preintervention, intervention, and postintervention groups

Variable	Preintervention, n = 947 (%)	Intervention, n = 278 (%)	Postintervention, n = 151 (%)	Total, n = 1,376 (%)	p value
Microorganism					
*Escherichia coli*	241 (58.78)	96 (55.17)	50 (49.02)	387 (56.41)	0.091
*Klebsiella pneumoniae*	70 (17.07)	35 (20.11)	22 (21.57)	127 (18.51)	
*Proteus mirabilis*	18 (4.39)	10 (5.75)	7 (6.86)	35 (5.1)	
*Pseudomonas aeruginosa*	24 (5.85)	8 (4.6)	2 (1.96)	34 (4.96)	
*Enterobacter cloacae*	6 (1.46)	4 (2.3)	4 (3.92)	14 (2.04)	
*Klebsiella oxytoca*	12 (2.93)	2 (1.15)	0 (0)	13 (1.9)	

**Table 5:** Antibiotics used for UTI treatment across the preintervention, intervention, and postintervention groups UTI: urinary tract infection

Variable	Preintervention, n = 947 (%)	Intervention, n = 278 (%)	Postintervention, n = 151 (%)	Total, n = 1,376 (%)	p value
Antibiotics					
Ceftriaxone	222 (52.48)	31 (38.27)	20 (44.44)	273 (49.73)	0.086
Piperacillin	78 (18.44)	19 (23.46)	10 (22.22)	107 (19.49)	
Cefazolin	38 (8.98)	8 (9.88)	4 (8.89)	50 (9.10)	
Meropenem	40 (9.46)	7 (8.64)	3 (6.67)	50 (9.10)	
Ertapenem	13 (3.07)	4 (4.94)	3 (6.67)	20 (3.64)	

Readmission due to UTI within 30 days remained stable across groups (p = 0.384). However, the median duration of antibiotic treatment and hospital length of stay significantly increased in the intervention and postintervention groups (p = 0.001 and p = 0.002, respectively) (Table 6). In-hospital mortality showed no significant variation among the groups (p = 0.488) (Table 3).

**Table 6:** Duration of antibiotic treatment and hospital stay across the preintervention, intervention, and postintervention groups

Variable	Preintervention, n = 947 (%)	Intervention, n = 278 (%)	Postintervention, n = 151 (%)	Total, n = 1,376 (%)	p value
Duration of antibiotic treatment, median (Q1-Q3) (days)	0 (0-3)	2 (0-4)	2 (0-4)	1 (0-3)	0.001

**Figure 3:**
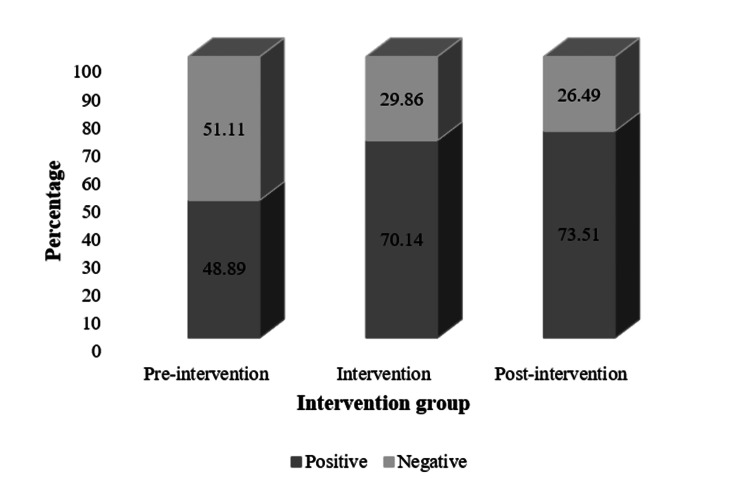
Urine culture results by intervention groups

The distribution of urine culture results across the study groups showed that, in the preintervention phase, the proportion of positive cultures was 48.89%. During the intervention phase, a significant increase in the proportion of positive cultures was observed, reaching 70.14%. This trend persisted in the postintervention phase, where positive cultures further increased to 73.51% (Figure 3).

**Figure 4:**
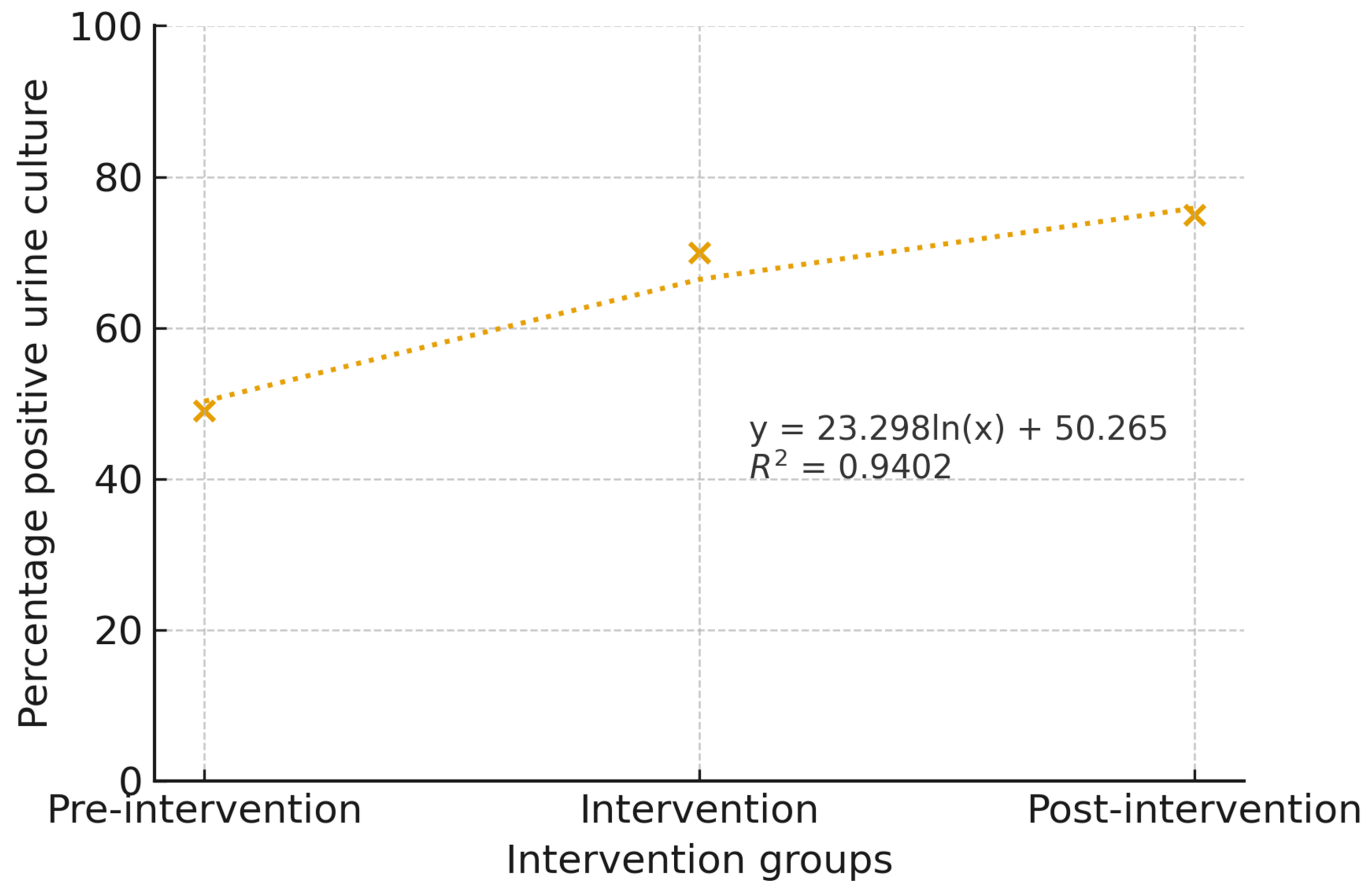
Logarithmic model

The logarithmic relationship in the model between the intervention and urine culture prescriptions indicates a 23.29% increase in the accuracy of UTI diagnosis when transitioning from the preintervention to the intervention phase. Given that the accuracy of urine culture prescriptions in the preintervention group was 50.27%, the shift to the postintervention phase led to a 23.29% improvement in accuracy. The coefficient of determination (R² = 0.9402) suggests that the intervention accounts for 94.02% of the variability in positive urine culture results (Figure 4).

**Figure 5:**
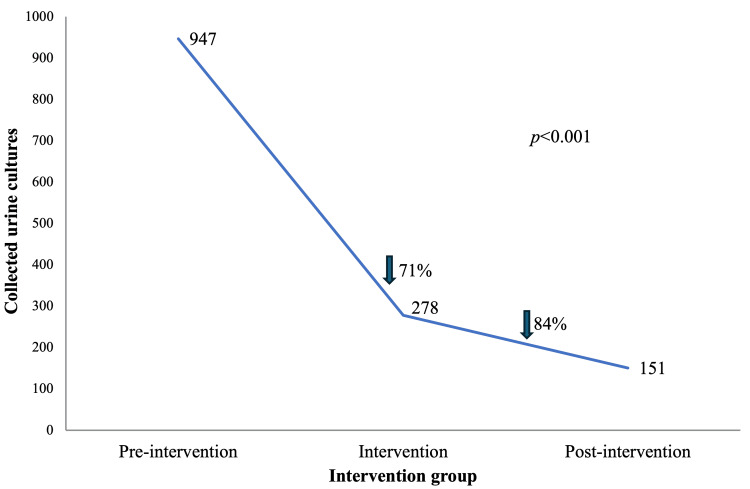
Trend in collected urine cultures by intervention groups

Similarly, the trend in the number of collected urine cultures showed a notable reduction following the implementation of the intervention, decreasing by 71% from the preintervention phase to the intervention phase, dropping from 947 to 278 cultures, and by 84% in the postintervention phase, reaching 151 collected cultures (Figure 5).

The costs associated with the ordering of urine cultures in the preintervention group were 43,508,968 Colombian pesos, while in the intervention group, they were 12,772,432 MVU, representing a 70.64% decrease in costs compared to preintervention costs. Maintaining the same in the postintervention group, the cost was 6,937,544 MVU, showing an 84.05% decrease in costs compared to the time before the intervention.

The costs associated with the days of hospitalization were calculated under the premise that each day has a cost of 341 MVU. In the preintervention phase, the total number of days of hospitalization was 1,938. During the intervention phase (during phase), 596 days were recorded, which represents a reduction of 1,342 days of hospitalization and a saving of 457,622 MVU. In the postintervention phase, 297 days were recorded, which is a difference of 299 days compared to the during phase, and an additional saving of 101,959 MVU. In total, the savings generated were 559,581 MVU.

## Discussion

In daily clinical practice, accuracy in the diagnosis of UTIs is essential. Therefore, a quasi-experimental study was conducted, analyzing 1,376 urine cultures. The results demonstrated a reduction in the screening of asymptomatic bacteriuria in patients who did not meet the treatment criteria, suggesting that the implemented strategy improved diagnostic accuracy for this condition. Furthermore, this intervention could help reduce unnecessary antibiotic use and decrease the need for hospitalization, aligning with a study conducted in Korea in 2015, which reported that 32% of asymptomatic bacteriuria cases received inappropriate treatment [14].

A significant reduction in the proportion of urine cultures performed was observed, with a decrease of 70.64% in the intervention group and 84.05% in the postintervention group. Similarly, a study conducted in 2020 demonstrated a reduction in urine culture processing following the implementation of a standardization program, which included specific criteria for ordering and the use of reflex urine culture analysis [15]. In Maryland, between 2011 and 2014, a urine culture protocol was implemented in an intensive care unit, limiting testing to cases with more than 10 leukocytes per field, excluding transplant and oncohematologic patients. This approach led to a reduction in the number of urine cultures ordered but, more importantly, resulted in changes in device use and a decrease in the rate of catheter-associated UTIs [16]. Daugherty et al. introduced a urine culture standardization strategy across multiple hospital departments, which led to a significant reduction in the number of urine cultures performed, decreasing the rate from 0.002 to -6.94 per 1,000 patient-days [15].

Similarly, an increase in the percentage of positive cultures was observed, with a 21.25% rise during the intervention phase and 24.62% in the postintervention phase compared to the preintervention phase. Similar results were reported in a study conducted at Hospital Pablo Tobón Uribe, in which the implementation of microbiological recommendations for urine culture requests in hospitalized patients led to an increase in culture positivity, rising from 29.7% in 1998 to 40.3% during 1999-2005. This increase was attributed to the active involvement of the laboratory in sample selection and the reduction of unnecessary urine cultures [17].

Our results indicate that, following the intervention, a reduction in the use of antibiotics such as ceftriaxone and meropenem was observed. This finding is consistent with another quasi-experimental study involving two general hospitals and 10 community clinics, where the implementation of specific criteria for urine culture processing led to a decrease in antimicrobial use, with an OR of 0.36 [18].

In terms of isolated pathogens, *E. coli* was the most prevalent, consistent with local epidemiological data, as it is the most commonly identified pathogen in the literature. A 2020 publication reports that *E. coli* is responsible for approximately 69%-73.9% of these infections [19]. Similarly, when antibiotics were used, ceftriaxone was the most frequently prescribed antibiotic for treatment, aligning with the primary antimicrobials recommended in parenteral regimens for the management of complicated UTIs in our country [5].

Our strategy proved safe, as readmission rates were similar across all groups. Additionally, no differences were observed in mortality. Regarding costs, a significant reduction was observed compared to the preintervention phase, particularly in costs associated with hospital length of stay and urine culture processing. These findings are consistent with a study published in 2023, which demonstrated that the removal of urinalysis and urine culture from preoperative checklists for cardiac surgery resulted in an estimated cost savings of $8,090.38, alongside a 50% reduction in antibiotic prescriptions for asymptomatic bacteriuria following the intervention [20]. In a previously published article, a calculation of the direct cost for processing unnecessary urine cultures was made, which indicated that considerable savings could be achieved if exclusion criteria based on the literature were applied [21].

Our study also demonstrated an improvement in the accuracy of UTI diagnosis following the intervention, reflected in an increased proportion of positive urine cultures. However, the associations between improved diagnostic accuracy and differences in antibiotic treatment duration and length of hospital stay should be interpreted cautiously, as these outcomes may reflect increased patient acuity or clinical complexity rather than a direct effect of improved diagnosis alone. It has been previously reported that successful diagnostic stewardship interventions are those that incorporate multifaceted and complementary approaches, both technical and educational, as demonstrated by the intervention described in our study [22]. The intervention was implemented using existing emergency department, laboratory, and Antimicrobial Stewardship Program personnel; however, additional time and manpower required during the initial implementation phase were not formally quantified. Given the quasi-experimental design and absence of randomization, causal inferences cannot be established, and residual confounding or secular trends may have influenced the observed associations. Finally, further multicentric studies are needed to confirm these findings and to facilitate broader implementation of standardized diagnostic stewardship strategies across different healthcare settings.

## Conclusions

The implementation of standardized urine culture protocols is essential to optimize the diagnosis of urinary tract infections, reduce unnecessary antibiotic use, and minimize associated healthcare costs. By refining diagnostic criteria, we not only improved the accuracy of UTI detection but also promoted more rational antimicrobial prescribing. Future research should focus on long-term outcomes, patient-centered benefits, and the adaptability of similar interventions in diverse clinical environments to further enhance the management of UTIs and antimicrobial stewardship efforts.

## References

[1] Mancuso G, Midiri A, Gerace E, Marra M, Zummo S, Biondo C (2023). Urinary tract infections: the current scenario and future prospects. Pathogens.

[2] Nagpal M, Chu J, Dobberfuhl A (2023). Mp39-20 cost of urinary tract infections with and without antibiotic resistance in the United States from 2012-2019. J Urol.

[3] Cardona Arias JA, Orrego Marin CP, Henao Mejia CP (2014). Prevalence of urinary tract infection, uropathogens, and antimicrobial susceptibility profile, Medellín 2011-2012. Acta Med Colomb.

[4] Sullivan KV, Morgan DJ, Leekha S (2019). Use of diagnostic stewardship practices to improve urine culturing among SHEA Research Network hospitals. Infect Control Hosp Epidemiol.

[5] Cortés JA, Cano Arenas N, Camero Blanco JD (2023). Clinical practice guideline for complicated urinary tract infection. Infectio.

[6] Flokas ME, Andreatos N, Alevizakos M, Kalbasi A, Onur P, Mylonakis E (2017). Inappropriate management of asymptomatic patients with positive urine cultures: a systematic review and meta-analysis. Open Forum Infect Dis.

[7] Sinawe H, Casadesus D (2023). Urine Culture. https://www.ncbi.nlm.nih.gov/books/NBK557569/.

[8] Kranz J, Bartoletti R, Bruyère F (2024). European Association of Urology guidelines on urological infections: summary of the 2024 guidelines. Eur Urol.

[9] Alkhawaldeh R, Abu Farha R, Abu Hammour K, Alefishat E (2022). Optimizing antimicrobial therapy in urinary tract infections: a focus on urine culture and sensitivity testing. Front Pharmacol.

[10] Ugalde-Espiñeira J, Bilbao-Aguirregomezcorta J, Sanjuan-López AZ, Floristán-Imízcoz C, Elorduy-Otazua L, Viciola-García M (2016). A program for optimizing the use of antimicrobials (PROA): experience in a regional hospital. [Article in Spanish]. Rev Esp Quimioter.

[11] Bou G, Cantón R, Martínez-Martínez L, Navarro D, Vila J (2021). Fundamentals and implementation of microbiological diagnostic stewardship programs. Enferm Infecc Microbiol Clin (Engl Ed).

[12] Cardoso CL, Muraro CB, Siqueira VL, Guilhermetti M (1998). Simplified technique for detection of significant bacteriuria by microscopic examination of urine. J Clin Microbiol.

[13] (2024). Statistical software for data science. https://www.stata.com/.

[14] Lee MJ, Kim M, Kim NH (2015). Why is asymptomatic bacteriuria overtreated? A tertiary care institutional survey of resident physicians. BMC Infect Dis.

[15] Dougherty DF, Rickwa J, Guy D, Keesee K, Martin BJ, Smith J, Talbot TR (2020). Reducing inappropriate urine cultures through a culture standardization program. Am J Infect Control.

[16] Epstein L, Edwards JR, Halpin AL (2016). Evaluation of a novel intervention to reduce unnecessary urine cultures in intensive care units at a tertiary care hospital in Maryland, 2011-2014. Infect Control Hosp Epidemiol.

[17] López JA, Cuartas MC, Molina OL (2006). Increment in positive urine cultures in a fourth level attention hospital. Colomb Assoc Infect Dis.

[18] De La Cadena E, Mojica MF, Castillo N (2020). Genomic analysis of CTX-M-Group-1-producing extraintestinal pathogenic E. coli (ExPEc) from patients with urinary tract infections (UTI) from Colombia. Antibiotics (Basel).

[19] Lee AL, Leung EC, Lee MK, Lai RW (2021). Diagnostic stewardship programme for urine culture: impact on antimicrobial prescription in a multi-centre cohort. J Hosp Infect.

[20] Winkler ML, Huang J, Starr J, Hooper DC, Paras ML, Letourneau AR, Shenoy ES (2023). If you don't test, they will not treat: impact of stopping preoperative screening for asymptomatic bacteriuria. Antimicrob Steward Healthc Epidemiol.

[21] Onderdonk AB, Winkelman JW, Orni-Wasserlauf R (1996). Eliminating unnecessary urine cultures to reduce costs. Lab Med.

[22] Advani S, Vaughn VM (2021). Quality improvement interventions and implementation strategies for urine culture stewardship in the acute care setting: advances and challenges. Curr Infect Dis Rep.

